# Supplementary material for: Three Models of Vaccination Strategies Against Cryptococcosis in Immunocompromised Hosts Using Heat-Killed Cryptococcus neoformans Δsgl1
Source: Front Immunol. 2022 May 9;13:868523. doi: 10.3389/fimmu.2022.868523 (PMC9124966; doi:10.3389/fimmu.2022.868523)
Supplement: Supplementary file 1 [file DataSheet_1.pdf]

frontiers

Supplementary Material

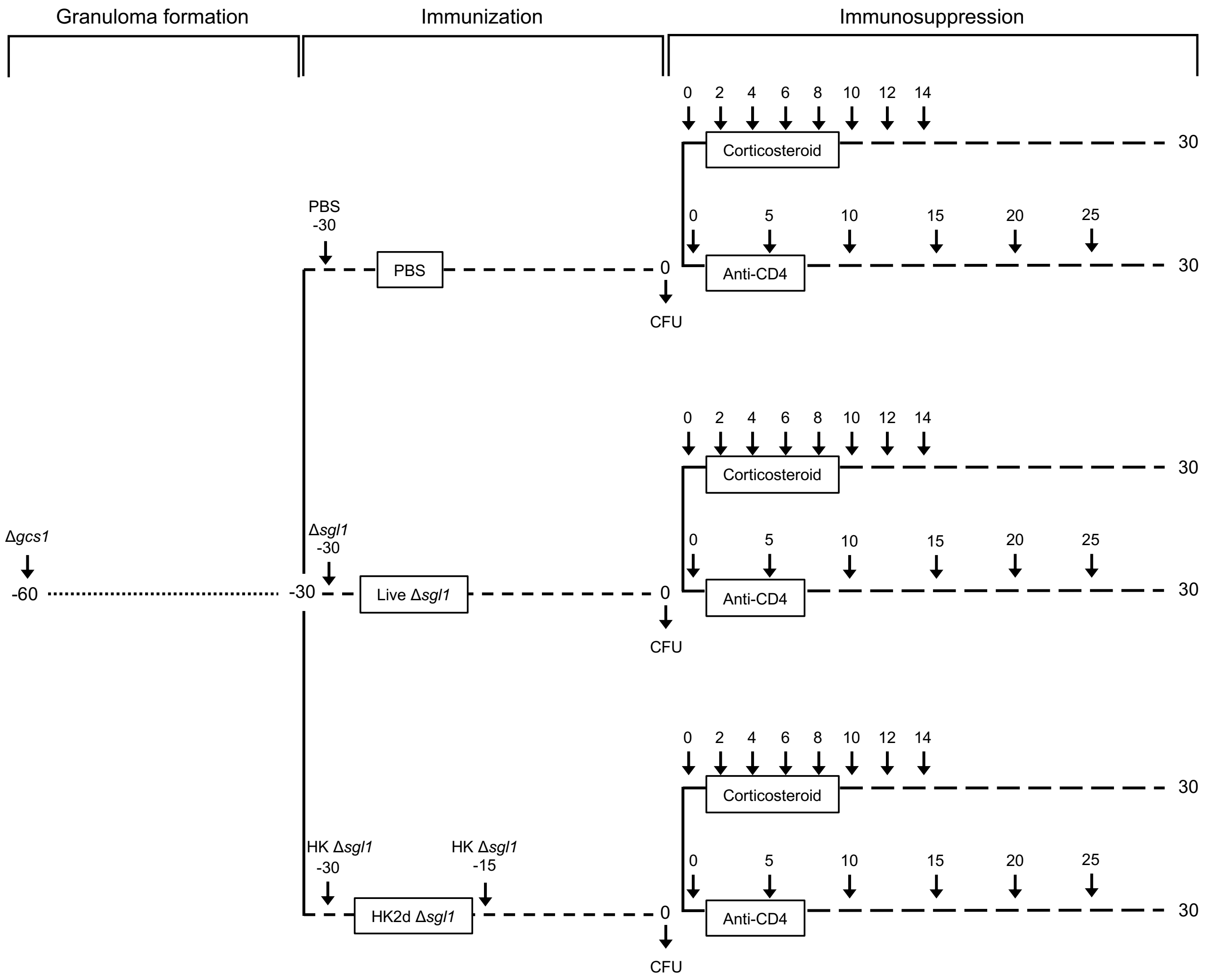

Supplementary Figure 1. Detailed experimental design schematic for Figure 7. CBA/J mice were intranasally infected with 5x10^5 *C. neoformans* Δ*gcs1* on day -60 to induce lung granuloma formation. After 30 days, mice were administered either 5x10^5 Live Δ*sgl1* or PBS on day -30 or two identical doses of 5x10^7 heat-killed (HK) Δ*sgl1* on days -30 and -15. Finally on day 0, 3 mice/group were sacrificed for lung fungal burden quantification prior to immunosuppression, while the remainder of the mice (n=10 mice/group/treatment) underwent continuous immunosuppressive treatment with either the corticosteroid cortisone acetate (CA) every 2 days for 14 days or anti-CD4 depletion antibody every 5 days to induce reactivation of the latent *C. neoformans* Δ*gcs1* yeast contained within lung granulomas and assessed for survival for 30 days.

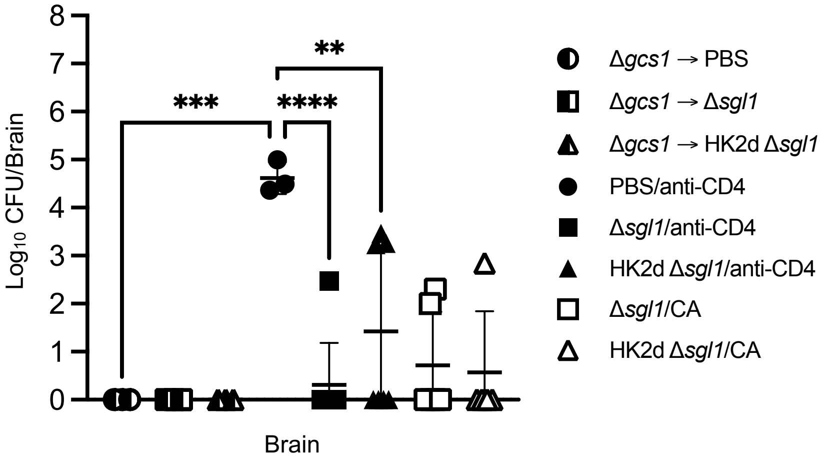

Supplementary Figure 2. Vaccination with either live or heat-killed (HK) *C. neoformans* Δ*sgl1* protects mice from extrapulmonary dissemination of reactivated yeast cells during immunosuppressive conditions. From Figure 7, endpoint brain fungal burden comparison in mice pre-immunosuppression on day 0 (Δ*gcs1* → PBS and Δ*gcs1* → Δ*sgl1*) (n=3 mice/group) and post-immunosuppression on day 30 for cortisone acetate (CA)-treated mice (Δ*sgl1*/CA and HK2d Δ*sgl1*/CA) (n=6-7 mice/group) and anti-CD4-treated mice (PBS/anti-CD4, Δ*sgl1*/anti-CD4, and HK2d Δ*sgl1*/anti-CD4) (n=9-10 mice/group). Graphed data represent the mean +/- SD. Significance was determined by an Ordinary one-way ANOVA using Tukey's multiple comparisons test for *P* value adjustment and is denoted as **, $P < 0.01$; ***, $P < 0.005$; ****, $P < 0.001$.
